# Efficacy of a short message service brief contact intervention (SMS-SOS) in reducing repetition of hospital-treated self-harm: randomised controlled trial

**DOI:** 10.1192/bjp.2023.152

**Published:** 2024-03

**Authors:** Garry John Stevens, Sandro Sperandei, Gregory Leigh Carter, Sithum Munasinghe, Trent Ernest Hammond, Naren Gunja, Anabel de la Riva, Vlasios Brakoulias, Andrew Page

**Affiliations:** School of Social Sciences, Western Sydney University, Penrith, New South Wales, Australia; Translational Health Research Institute, Western Sydney University, Penrith, New South Wales, Australia; School of Medicine and Public Health, University of Newcastle, Callaghan, New South Wales, Australia; Nepean Clinical School, Faculty of Medicine and Health, University of Sydney, Penrith, New South Wales, Australia; Westmead Clinical School, Faculty of Medicine and Health, University of Sydney, Westmead, New South Wales, Australia; and Emergency Department, Westmead Hospital, Westmead, New South Wales, Australia; Westmead Hospital, Western Sydney Local Health District, Westmead, New South Wales, Australia

**Keywords:** Self-harm, aftercare, prevention, out-patient treatment, randomised controlled trial

## Abstract

**Background:**

Hospital-treated self-harm is common and costly, and is associated with repeated self-harm and suicide.

**Aims:**

To investigate the effectiveness of a brief contact intervention delivered via short message service (SMS) text messages in reducing hospital-treated self-harm re-presentations in three hospitals in Sydney (2017–2019), Australia. Trial registration number: ACTRN12617000607370.

**Method:**

A randomised controlled trial with parallel arms allocated 804 participants presenting with self-harm, stratified by previous self-harm, to a control condition of treatment as usual (TAU) (*n* = 431) or an intervention condition of nine automated SMS contacts (plus TAU) (*n* = 373), over 12 months following the index self-harm episode. The primary outcomes were (a) repeat self-harm event rate (number of self-harm events per person per year) at 6-, 12- and 24-month follow-up and (b) the time to first repeat at 24-month follow-up.

**Results:**

The event rate for self-harm repetition was lower for the SMS compared with TAU group at 6 months (IRR = 0.79, 95% CI 0.61–1.01), 12 months (IRR = 0.78, 95% CI 0.64–0.95) and 24 months (IRR = 0.78, 95% CI 0.66–0.91). There was no difference between the SMS and TAU groups in the time to first repeat self-harm event over 24 months (HR = 0.96, 95% CI 0.72–1.26). There were four suicides in the TAU group and none in the SMS group.

**Conclusions:**

The 22% reduction in repetition of hospital-treated self-harm was clinically meaningful. SMS text messages are an inexpensive, scalable and universal intervention that can be used in hospital-treated self-harm populations but further work is needed to establish efficacy and cost-effectiveness across settings.

Hospital-treated self-harm is common,^1^ costly^2^ and linked to several mental health and social problems, including increased suicide risk,^1^ long-term comorbid mental disorders (e.g. anxiety, mood, substance use and personality disorders) and psychological distress.^3^ There is no clearly agreed definition of ‘deliberate self-harm’ in Australia and New Zealand,^4^ but in the UK ‘self-harm’ is principally defined as ‘intentional self-poisoning or injury, irrespective of the apparent purpose’ in the National Institute for Health and Care Excellence (NICE) Guidelines.^5^ A recent systematic review reported that self-poisoning (median: 90%) and self-cutting (median 10.5%) were the most commonly used methods of self-harm among those presenting to hospital.^1^ In Australia during the period 2016–2017, self-harm accounted for 7% (33 100) of hospital admissions for injury, with this subset principally consisting of self-poisoning (83%) and self-injury by a sharp object (12%).^6^ The most relevant suicidal behaviour outcomes relating to the present study are repetition of non-fatal self-harm (16% at 1 year) and suicide (1.6% at 1 year; 3.9% at 5 years).^1^

People who engage in self-harm typically have low adherence to treatment in aftercare^7^ and so alternative interventions are needed that can be easily delivered, are acceptable to patients and clinically effective. A systematic review of the effectiveness of brief contact interventions (BCIs) for suicidal behaviour reported a non-significant reduction for the binary outcomes of any repeated self-harm (pooled odds ratio OR = 0.87 (95% CI 0.74–1.04) and suicide (pooled OR = 0.58, 95% CI 0.24–1.38) for all BCIs combined.^8^ For three studies using a postcard or written letter as the BCI, the meta-analysis showed a significant reduction in the self-harm event rate (i.e. number of repetitions per person per year) (incidence rate ratio IRR = 0.66, 95% CI 0.54–0.80).^8^ Using a wider definition of BCIs, i.e. ‘brief acute care’ interventions (e.g. including safety planning plus brief contact), a later systematic review reported a significant benefit for the binary outcome of any subsequent ‘suicide attempts’ (pooled OR = 0.69, 95% CI 0.53–0.89).^9^ The mechanisms of action by which BCIs might reduce repetition of hospital-treated self-harm are unproven; however, previous studies have proposed that BCIs may increase an individual's sense of ‘social connectedness’ – the perceived quality and size of their social network – potentially supporting reduced risk.^7^ Vaiva et al (2006) found that the provision of contact interventions may have facilitated access to clinical services (cited in^8^).

## SMS-supported aftercare

Text messages delivered by a short message service (commonly known as SMS messages or SMS), rather than via postcards or letters, may be an effective way to communicate with patients and influence health behaviours. Pilot studies have highlighted the acceptability and feasibility of SMS in populations at risk of suicide.^10,11^ A systematic review of mobile phone and web-based text messaging interventions for people with mental health conditions found that 35 out of 36 studies indicated that respondents felt positive in receiving an SMS.^12^ Questionnaires and satisfaction surveys showed that text messaging was acceptable and improved health outcomes, including treatment and appointment attendance.^12^ Although a range of BCIs have been evaluated, the only previous randomised controlled trial (RCT) to have examined the effects of SMS messaging in self-harm populations was conducted among US military personnel,^13^ finding no significant effects on rates of suicidal ideation, subsequent suicide attempts or emergency department visits. Similarly, an RCT conducted in US veteran populations using email delivery of a BCI showed no benefit on all-cause hospital re-presentations and self-reported suicide attempts or psychiatric hospital admissions.^14^

## Aims

This study aimed to evaluate the effectiveness of a schedule of SMS text messages (plus treatment as usual (TAU)) compared with TAU among individuals with hospital-treated self-harm on: (a) the primary outcomes of (i) number of repeat hospital-treated self-harm events per person per year at 6, 12 and 24 months post-index self-harm episode and (ii) time to first hospital-treated self-harm repeat at 24 months; and (b) the secondary outcomes of (i) any self-harm event at 6, 12 and 24 months post-index self-harm episode and (ii) the number of suicides at 24 months.

## Method

The design and protocol of the SMS for Self-harm or Suicide (SMS-SOS) study has been previously described.^15^ A summary is presented below to outline key details and important contextual information.

### Setting

This study was conducted in three tertiary teaching hospitals in two local health districts (LHDs) in New South Wales (NSW), Australia: Western Sydney LHD (Westmead Hospital and Blacktown Hospital) and Nepean Blue Mountains LHD (Nepean Hospital).

### Inclusion and exclusion criteria

Patients presenting with self-harm to hospital emergency departments were initially identified as potentially eligible participants by mental health clinicians (e.g. psychiatrists, psychiatry trainees, psychologists and specialist nursing staff) during standard clinical mental health assessments.

Other inclusion criteria were that participants were 16 years or older, had a good understanding of written and spoken English and an active Australian mobile phone number. Exclusion criteria were: declined to participate, did not have an active Australian mobile phone number, did not have a fixed Australian address or were unable to provide informed consent.

### Study design

**Fig. 1:**
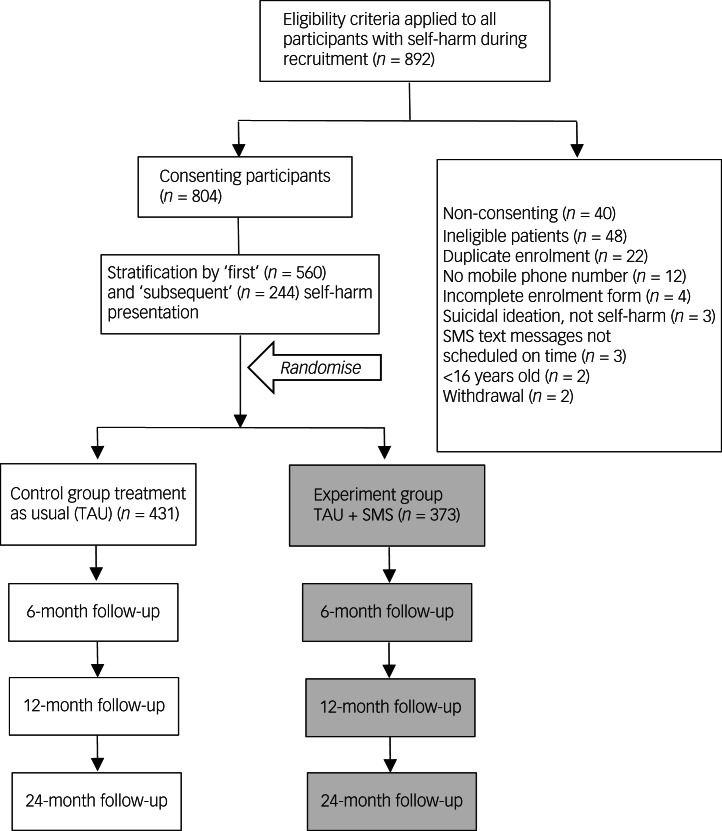
CONSORT diagram of flow through the study. SMS, short message service.

A multi-centre, parallel RCT was conducted across three large public hospitals in Australia. Patient enrolment occurred between 3 October 2017 and 6 December 2019 and ceased when the required sample size was attained. The study used a single-consent Zelen design for the SMS condition (Supplementary Note 1, available at https://dx.doi.org/10.1192/bjp.2023.152). The study compared a control group (TAU) with an intervention group (SMS plus TAU) (Fig. 1).

### Randomisation

Since there are substantially different self-harm repetition rates for those with and without a history of self-harm, participants were first stratified (first ever hospital-treated self-harm versus any subsequent episode), then randomly assigned and enrolled into the study. Randomisation was done within strata, with researchers using random permuted blocks of size 6 (to balance allocation) to either the SMS or TAU condition using computer-generated randomisation.

Clinicians enrolled eligible participants into either the SMS or TAU conditions based on allocation information obtained from numbered envelopes. The allocating clinician selected the next sequentially numbered opaque envelope for each participant to reveal the intervention allocation. Study researchers within each hospital emergency department routinely confirmed the placement of research materials and that the Allocation Information Sheets within envelopes matched the randomised number sequence, to ensure the fidelity of the allocation.

### Procedures

For participants allocated to the SMS condition, clinicians explained that the study involved receiving text messages for up to 12 months after leaving the hospital and that all study data were anonymised. Clinicians provided patients in the SMS condition with a Participant Information Sheet and obtained written consent. Those who declined consent were excluded from the trial. Study enrolment involved clinicians attaching participant identification information to an Allocation Information Sheet, which was then entered into a study database in preparation for prospective data linkage of re-presentations over the follow-up period. A manual check of enrolled patients, augmented by a self-harm identification ‘flag’ established in the electronic medical record (EMR) in two of the participating hospitals (Supplementary Note 2), was also conducted to prevent inadvertently re-enrolling existing participants on any re-presentation events.

### Control and intervention conditions

The TAU control condition was not standardised, and included the usual hospital treatment for self-harm, including emergency department physical care (e.g. treatment for drug overdose, wound management, etc.), psychiatric consultation, medication where necessary and aftercare, including mental health outreach services (e.g. follow-up assessment and counselling) and primary care. Patient aftercare varies for each individual but usually consists of hospital and/or community mental health service contact within 24 h of discharge and a recommended programme of care, including clinical reassessment, mental health and psychological therapies, drug and alcohol services and/or referral to a general practitioner. Participants allocated to the TAU group were not required by clinicians to provide written consent, as per the Zelen single-consent study design (Supplementary Note 1) that was approved by the ethics committee.

The SMS condition included a schedule of nine text messages over a 12-month follow-up period plus TAU. The SMS content was developed using detailed feedback from a community panel of lived experience consultants. The SMS messages were personalised with the name of the receiving participant and were sent on identical schedules, based on the week of hospital discharge. Participants were aware that the messages were automated and did not support replies. Nine text messages were sent to those in the SMS condition at months 1, 2, 3, 4, 5, 6, 8, 10 and 12 post-discharge. This schedule was similar to that employed in the previous ‘Postcards from the EDge’ studies in Australia.^16^ Each SMS used one of three different message formats, on a rotating schedule, to maintain some novelty (Supplementary Boxes 1–3). These expressed concern for the participant's well-being and encouraged them to telephone clinical crisis teams and mental health support services, including Lifeline and the NSW Health ‘Mental Health Line’, if needed.

### Outcome measures

The primary outcome measures (at 6, 12 and 24 months post-index self-harm episode) were: (a) the number of hospital self-harm repetitions per person (repetition event rate) and (b) the time to first self-harm re-presentation at 24 months. The secondary outcomes were (a) the difference in proportions for any self-harm re-presentation (at 6, 12 and 24 months post-index self-harm episode) and (b) the number of suicides.

Subsequent self-harm re-presentations to an emergency department in any public hospital in NSW for all participants was determined via probabilistic record linkage with the NSW Emergency Department Data Collection (EDDC) and Admitted Patient Data Collection (APDC) (conducted by the NSW Centre for Health Record Linkage, CHeReL). Self-harm events in any emergency department were identified using a combination of two diagnostic fields available in the EDDC database: ‘main diagnosis’ and ‘presenting problem’. Self-harm presentations included those who were formally admitted to hospital, as well as those treated only in the emergency department. The ‘main diagnosis’ field in the EDDC was coded using the Systematized Nomenclature of Medicine Clinical Terms (SNOMED CT) classification system (Supplementary Note 2). Its ‘presenting problem’ field allows selection from approximately 280 pre-formatted case descriptions; cases of self-harm were defined where the emergency department presentation was coded as ‘MH – Self-Harm’. Self-harm events resulting in hospital admission were also enumerated via the APDC, based on the ICD-10 codes relating to self-harm (X60–X84) in any of the 51 diagnostic fields. Self-harm cases were also included from a recently introduced EMR field specifically asking emergency department clinicians if the reason for mental health (MH) referral involved self-harm. Cases of mortality were enumerated via linkage to the Australian Bureau of Statistics Cause of Death Unit Record File. Follow-up data were available for the period October 2017 to March 2022, allowing for an assessment of event rates at 6 months, 12 months and 24 months after the index presentation.

### Other study factors

Clinical and demographic patient information was also extracted from clinical records to evaluate potential imbalances at randomisation, and included previous clinical diagnoses, sex, birth date, marital status at enrolment and country of birth.

### Sample size calculation

A total sample size of 796 participants was proposed (398 participants in the SMS condition and 398 in the control group), as previously described.^15^ This assumed an incidence rate ratio (IRR) based on event rates of IRR = 0.66 (see^17^) for the SMS condition compared with TAU, with a 5% significance level, 80% power and 10% adjustment for correlation of individuals within hospitals. It was also assumed that the 12-month re-presentation event rate for self-harm was ~15% in the TAU group,^15^ or a probability of survival of 0.85. A median survival time (time to first repetition) in the TAU condition of 4.3 years was also assumed.^16^ For assessing differences in median time to first repetition, assuming a median survival time in the TAU group of 73.5 days^16^ and a similar relative difference in event rates between SMS and TAU conditions as above (IRR = 0.66),^17^ the estimated median survival time in the SMS condition was 121.8 days. This anticipated difference in time to first re-presentation between SMS and TAU conditions requires a total sample size of 138 participants (69 in the SMS condition and 69 in the TAU condition), with 5% significance, 80% power and 10% adjustment for intra-class correlation within hospitals.

### Statistical analyses

Participant characteristics were summarised using mean, standard deviation, and absolute and relative frequencies. The TAU and SMS groups were first compared using a multivariate logistic regression to assess any imbalances at randomisation associated with potential confounding variables (sex, age, country of birth, main language spoken at home, marital status, presenting hospital and first or subsequent presentation) in the allocation to treatment groups.

All outcome analyses were based on the intention-to-treat principle, defined by the allocation status of participants. For the primary outcomes the differences in the self-harm repetition event rates (self-harm events per person per year) between the SMS and TAU conditions at 6, 12 and 24 months were examined using a Poisson regression model to estimate the IRRs, and absolute risk differences based on incidence proportions. Data overdispersion was assessed using the Cameron and Trivedi overdispersion test,^18^ resulting in a non-significant result (*P*-values varying from 0.08 to 0.11). A sensitivity analysis was also conducted to investigate differences in the incidence of subsequent self-harm between the SMS and TAU conditions at 6, 12 and 24 months using a Hurdle negative binomial regression model, which accounted for possible overdispersion and zero inflation in the data (Supplementary Table 1). The time to first re-presentation was assessed using a Cox proportional hazards model. Given the proportionality assumption for the model,^19^ only one model was specified for the 24-month follow-up period. All analyses were performed in R, version 4.3.2 for Linux Ubuntu 20.04.6 LTS, using packages ‘survival’^20^ and ‘pscl’.^21^

### Ethical approvals and trial registration

Participants in the SMS condition were informed that they could withdraw from the study at any time without prejudice. The authors assert that all procedures contributing to this work comply with the ethical standards of the relevant national and institutional committees on human experimentation and with the Helsinki Declaration of 1975, as revised in 2008. All procedures involving human participants/patients were approved by the NSW Ministry of Health and Western Sydney Local Health District's Human Research Ethics Committee (protocol number: HREC/16/WMEAD/336). The whole study complied with the Australian National Health and Medical Research Council's National Statement on Ethical Conduct in Human Research (2007) (updated in 2018). Site-specific approval was granted by Nepean Blue Mountains Local Health District's Research Governance Office (SSA/16/Nepean/170). The study's clinical trial registration number is ACTRN12617000607370 (registered with https://www.anzctr.org.au/).

## Results

### Study population

During the recruitment period the recruiting clinicians identified *n* = 892 hospital-treated self-harm patients, *n* = 88 of whom did not consent or were ineligible to participate in the study (Fig. 1). Thus, a total of 804 eligible participants were enrolled, with stratification by history of self-harm resulting in *n* = 560 with no history and *n* = 244 with a history of self-harm. There were 431 participants in the TAU (control) group and 373 in the SMS (intervention) group.

### Participant characteristics and randomisation

The participants were predominately female, aged 16–24 years, Australian-born, spoke English at home and had never been married (Table 1). Approximately 30% had a history of self-harm, which is consistent with prevalence estimates within Australian hospitals.^3^ Recruitment by site reflected the relative size of clinical service delivery of the three hospitals: Westmead (*n* = 317, 39%), Blacktown (*n* = 263, 33%) and Nepean (*n* = 224, 28%). There were no imbalances after randomisation for selected demographic factors, previous self-harm or hospital site, indicating there was no requirement to adjust analyses for these characteristics (Table 1). Stratification by history of self-harm (because of the strong association with repeated self-harm events) also ensured that no adjustment to analyses was required for this characteristic. The 88 participants who did not meet eligibility criteria and were excluded from the study did not differ from study participants in relation to hospital site distribution (χ^2^ = 4.52, *P* = 0.104) and had a marginally lower propensity for any repeat self-harm at 24-month follow-up (χ^2^ = 3.63, *P* = 0.057).

**Table 1:** Demographic and clinical characteristics of participants by group

	Treatment as usual group (*n* = 431), *n* (%)	SMS intervention group (*n* = 373), *n* (%)
Sex		
Male	153 (35.5)	131 (35.1)
Female	278 (64.5)	242 (64.9)
Enrolment age, years		
16–24	192 (44.5)	165 (44.2)
25–44	148 (34.3)	141 (37.8)
45+	91 (21.1)	67 (18.0)
Country of birth		
Australia	338 (78.4)	281 (75.3)
Other	92 (21.3)	87 (23.3)
n.a.	1 (0.2)	5 (1.3)
Language spoken at home		
English	403 (93.5)	345 (92.5)
Other	25 (5.8)	22 (5.9)
n.a.	3 (0.7)	6 (1.6)
Marital status		
Never married	291 (67.5)	243 (65.1)
Married	93 (21.6)	85 (22.8)
Separated/widowed/divorced	45 (10.4)	39 (10.5)
n.a.	2 (0.5)	6 (1.6)
Presentation history		
First presentation	297 (68.9)	263 (70.5)
Subsequent presentation	134 (31.1)	110 (29.5)
Presenting hospital		
Westmead	169 (39.2)	148 (39.7)
Blacktown	140 (32.5)	123 (33.0)
Nepean	122 (28.3)	102 (27.3)

n.a., data not available; SMS, short message service.

### Primary outcomes

**Fig. 2:**
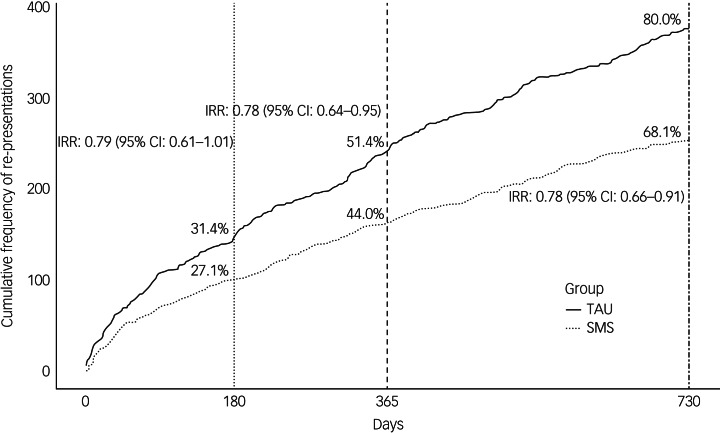
Cumulative frequency of re-presentations for hospital-treated self-harm by groups at 6-month, 12-month and 24-month follow-up. TAU, treatment as usual; SMS, short message service; IRR, incidence rate ratio.

Event rates for hospital-treated repeat self-harm were lower for the SMS compared with the TAU group at 6 months (relative risk reduction (RRR) = 21%, IRR = 0.79, 95% CI 0.61–1.01), 12 months (RRR = 22%, IRR = 0.78, 95% CI 0.64–0.95) and 24 months (RRR = 22%, IRR = 0.78, 95% CI 0.66–0.91) (Fig. 2). This represented 123 fewer self-harm re-presentations over the 24-month follow-up period.

A *post hoc* subgroup analysis by sex suggested higher re-presentation event rates among females than males and a stronger effect of the SMS intervention among females than males at 24-month follow-up compared with the TAU group (Table 2). For males, there was no statistical difference in the event rates between the SMS and TAU groups (RRR = −6%, IRR = 1.06, 95% CI 0.71–1.56). For females, repeat self-harm was lower in the SMS group compared with the TAU group (RRR = 27%, IRR = 0.73, 95% CI 0.61–0.87).

**Table 2:** Frequency of re-presentations at 6-month, 12-month and 24-month follow-up, by sex

	Males		Females		Total	
Number of re-presentations	TAU group (*n* = 153), *n* (%)	SMS group (*n* = 131), *n* (%)	TAU group (*n* = 278), *n* (%)	SMS group (*n* = 242), *n* (%)	TAU group (*n* = 431), *n* (%)	SMS group (*n* = 373), *n* (%)
At 6-month follow-up						
0	139 (90.8)	113 (86.3)	233 (83.8)	206 (85.1)	372 (86.3)	319 (85.5)
1	9 (5.9)	12 (9.2)	28 (10.1)	23 (9.5)	37 (8.6)	35 (9.4)
2	3 (2.0)	4 (3.1)	12 (4.3)	5 (2.1)	15 (3.5)	9 (2.4)
3	2 (1.3)	1 (0.8)	2 (0.7)	4 (1.7)	4 (0.9)	5 (1.3)
4+	0 (0.0)	1 (0.8)	3 (1.1)	4 (1.7)	3 (0.7)	5 (1.3)
At 12-month follow-up						
0	131 (85.6)	107 (81.7)	212 (76.3)	191 (78.9)	343 (79.6)	298 (79.9)
1	16 (10.5)	18 (13.7)	40 (14.4)	24 (9.9)	56 (13.0)	42 (11.3)
2	3 (2.0)	4 (3.1)	13 (4.7)	14 (5.8)	16 (3.7)	18 (4.8)
3	0 (0.0)	1 (0.8)	4 (1.4)	5 (2.1)	4 (0.9)	6 (1.6)
4+	3 (2.0)	1 (0.8)	9 (3.2)	8 (3.3)	12 (2.8)	9 (2.4)
At 24-month follow-up						
0	123 (80.4)	105 (80.2)	199 (71.6)	178 (73.6)	322 (74.7)	283 (75.9)
1	21 (13.7)	17 (13.0)	41 (14.7)	29 (12.0)	62 (14.4)	46 (12.3)
2	4 (2.6)	4 (3.1)	21 (7.6)	13 (5.4)	25 (5.8)	17 (4.6)
3	1 (0.7)	1 (0.8)	4 (1.4)	8 (3.3)	5 (1.2)	9 (2.4)
4+	4 (2.6)	4 (3.1)	13 (4.7)	14 (5.8)	17 (3.9)	18 (4.8)

TAU, treatment as usual; SMS, short message service.

For the time to first re-presentation, there was no difference between the SMS and TAU groups over the 24-month follow-up period (HR = 0.96, 95% CI 0.72–1.26) (Supplementary Figure 1).

### Secondary outcomes

There was no difference in the binary end-point of any repeated self-harm between the TAU and SMS groups at 6-month (TAU = 13.7%, 95% CI 4.7–22.6% *v.* SMS = 14.5%, 95% CI 4.9–24.1%), 12-month (TAU = 20.4%, 95% CI 11.9–29% *v.* SMS = 20.1%, 95% CI 10.9–29.3%) or 24-month (TAU = 25.3%, 95% CI 17–33.5% *v.* SMS = 24.1%, 95% CI 15.2–33.1%) follow-up.

There were 16 deaths among the 804 participants over the 24-month follow-up period, of which four were suicides. All the cases of suicide occurred in the TAU group.

## Discussion

This study showed some effectiveness on repeat self-harm events, using an aftercare text messaging intervention for people presenting for hospital treatment of self-harm. Event rates for self-harm re-presentations were lower among those who received SMS messages plus TAU compared with those who received TAU, with a relative risk reduction of 22% at 24-month follow-up, equating to 123 fewer self-harm re-presentations in the SMS group compared with the TAU group. A relative risk reduction of 22% represents a clinically important difference given the absolute number of self-harm re-presentations that would be prevented with this automated and low-cost intervention. For example, a 22% relative risk reduction for all self-harm re-presentations would equate to 300 fewer self-harm re-presentations per year across the three largest Western Sydney hospitals in the study, a considerable opportunity cost for service providers. Reduced episodes were observed in females in the SMS group – principally those with more frequent re-presentations – in *post hoc* analysis. Suicide deaths were infrequent, as expected, even in this ‘high-risk’ clinical population. Although there were no suicide deaths in the SMS group, this beneficial result cannot be regarded as definitive.

Not all measures of representation of self-harm benefited from the SMS-SOS intervention. There was no difference in the time to first re-presentation event; no difference in the proportion of individuals who repeated self-harm (any event) as a secondary outcome; and no observed effect among males in a *post hoc* analysis. The current study provides an important demonstration of the effectiveness of SMS-based aftercare interventions, deployed from the general hospital, on some measures of non-fatal repeat self-harm. It is the largest study of an SMS contact intervention to date and its findings are broadly similar to those observed with other BCI formats (including postcards, letters and phone calls). Milner et al^8^ found that BCIs were also more effective at reducing the number of repeat self-harm events per person per year, with a non-significant benefit for the difference in proportions of individuals who had any repeat self-harm event, following the index admission. Other studies have demonstrated that most people who present to hospital following self-harm will not have any repeat events after 12 months^1^ and studies used *post hoc* analysis to demonstrate that the beneficial effects of BCIs might be best seen in females with multiple repeat self-harm events.^16^

The possible mechanisms of action for BCIs on self-harm have not yet been demonstrated. Milner et al found that the most commonly suggested mechanisms in BCI studies related to increased social support and suicide prevention literacy.^7^ Perceived social support has been associated with reduced stress and re-attempt rates in the first 12 months post-discharge.^22^ The nature of BCI contacts may also change attitudinal barriers regarding the care process, which people who self-harm often perceive as negative.^7^ We might speculate that the conveyed message that health providers care about their well-being and that there is an ongoing offer of help may differentially resonate with females likely to have multiple self-harm repetition events, compared with other subgroups. Larger trials stratified by sex and self-harm history, and qualitative studies, are needed to determine subgroup effects and their underlying mechanisms.^8^

### SMS schedule and automation

A notable feature of the SMS aftercare trial in the US military^13^ was its use of early contact points at day 1 and week 1 post-discharge (for discharged samples) and on participants’ birthdays. For those discharged from hospital, the risk of a repeat attempt is highest in the period immediately after discharge; one in three re-attempts occur within the first 30 days.^23^ Therefore SMS contacts within 1 month of the index admission may have a role in reducing risk and enhancing programme engagement during this key period. The current study adopted the schedule of an Australian ‘Postcards from the EDge’ trial^16^ to allow comparison, but future trials of the SMS-SOS programme could test the inclusion of earlier contact points.^10,13^

It is important to consider the current findings in the context of the wider evidence base regarding suicide prevention and self-harm reduction. Innovative approaches are needed for those carrying out self-harm, who are a difficult-to-treat population, have low adherence to treatment regimens over time and a relatively high likelihood of self-harm recurrence.^8^ Vaiva et al (2006) highlighted the emerging delineation between ‘intensive’ and ‘connectedness’ care approaches for this population (cited in^8^). Intensive approaches include formal therapy such as cognitive–behavioural therapy or dialectical behaviour therapy, which have shown reductions in self-harm recurrence of up to 50% in clinical trials.^24^ However, such treatments are resource intensive, require specialist training and are not feasible in many environments. In contrast, BCIs can be delivered at low cost, maintain long-term engagement and can support re-contact with clinical services when needed, either implicitly or through direct invitation. SMS is one of the few BCI formats that can be fully automated, incurring almost no ongoing cost to clinician time or service systems. They also provide a ready platform for self-management using psychoeducation tools, and direct contact with helplines and mental health services. The latter can also be triggered via ecological momentary assessments (e.g. physical activity, mobility and sleep indicators as proxy markers of mental health).^25^

### Limitations and strengths

There are a number of methodological limitations when considering the findings of the current study. First, it is possible that the study was underpowered to detect differences in self-harm re-presentations between the SMS intervention and TAU groups because of lower intervention effect sizes in this study (IRR = 0.78) compared with the pooled estimates of previous studies (IRR = 0.66) that were used in the power calculations. The weaker rate ratio likely reflects the different contexts, populations and modality of the intervention, in that the ‘Postcards from the EDge’ study^16^ and subsequent ‘Postcards from Persia’ and ‘Postcards from Christchurch’ studies (cited in^8^) used physical mail contacts and were conducted in single hospitals with different catchment populations, compared with the more complex setting of the current study, involving multiple hospital settings with competing priorities. Additionally, the relative difference between the SMS intervention and TAU groups reflects a small absolute difference in self-harm case numbers at 24-month follow-up (*n* = 19).

Second, there may have been selection bias in the intervention group. In a single-consent Zelen design, where only the intervention group participants are required to consent to the intervention, a number of potential participants will decline (Supplementary Note 1). Some individuals randomised to SMS declined to consent, as can be seen by the difference in the number of participants in the TAU group (*n* = 431) and the SMS group (*n* = 373). If those who declined to consent to the SMS intervention were more likely or less likely, on average, to have multiple repeat self-harm events than those who consented to the SMS condition, then this may have affected the results of the study to decrease or increase the observed effect size respectively. A further issue relates to patients with emergency department-identified self-harm who do not receive a subsequent mental health assessment. It is a requirement in Western Sydney hospitals that all such patients are referred for in-patient mental health assessment. Emergency department staff estimate that non-referral rates are very low (<1%) (Supplementary Note 2). The assessment rate from referrals is over 80%, with patient absconding the primary reason for non-assessment. If this group of non-assessed patients were more or less likely, on average, to have multiple repeat self-harm, then this could also potentially increase or decrease the effect size.

Third, there is potential measurement bias in the enumeration of hospital-treated repeat self-harm. Measurement bias in defining self-harm at baseline is unlikely, as enrolment into the study as a case of self-harm was based on a clinician assessment following presentation to the emergency department in one of the participating hospitals. Repeat self-harm events were enumerated based on routinely coded information in hospital data, a potential source of misclassification in the outcome. However, the modified approach to include other diagnostic fields ensured a more complete enumeration of cases than using ICD-10 and SNOMED-CT codes alone, and event rates were similar to other reported estimates.^1,16^ It is also possible that a small number of repeat self-harm events presented to hospitals in other states, as follow-up data linkage was restricted to the NSW services.

Fourth, a set of potential confounding variables, based on routinely collected demographic and health service variables, were distributed via randomisation, without any imbalance being demonstrated. There are likely other potential confounders that have not been assessed in the current study. However, randomisation (with sufficiently large sample size) accounts for both measured and unmeasured confounding, and potential confounders would need to be differentially distributed between intervention and control groups such that one group had higher likelihood of re-presentation for self-harm than another group.

Despite these limitations, the current study demonstrates the effectiveness of SMS in reducing self-harm re-presentation event rates. A key strength is the randomised study design, based on an approach to all cases of self-harm meeting the study criteria in the three main public hospitals in Western Sydney, with subsequent self-harm case enumeration based on data linkage with all NSW public hospitals over a 2-year follow-up period. Additionally, stratification by history of previous self-harm (because of the strong association with repeated self-harm events) ensured that no adjustment to analyses was required for this characteristic. The findings would be cautiously generalisable to other hospital settings in New South Wales, and the automated and low-cost nature of SMS suggests that it is feasible that this could be scaled up more widely.

## Supporting information

Stevens et al. supplementary material 1 Stevens et al. supplementary material

Stevens et al. supplementary material 2 Stevens et al. supplementary material

Stevens et al. supplementary material 3 Stevens et al. supplementary material

Stevens et al. supplementary material 4 Stevens et al. supplementary material

Stevens et al. supplementary material 5 Stevens et al. supplementary material

## Data Availability

Unless required by law, study data will not be provided to third parties. This is due to several reasons: the importance of complying with hospital ethics and governance regulations, to maintain research participant confidentiality and to ensure the data are not misinterpreted by people unfamiliar with hospital procedures, databases and terminologies.
