# Supplementary material for: Efficacy of a short message service brief contact intervention (SMS-SOS) in reducing repetition of hospital-treated self-harm: randomised controlled trial
Source: Br J Psychiatry. 2024 Mar;224(3):106–13. doi: 10.1192/bjp.2023.152 (PMC10884824; doi:10.1192/bjp.2023.152)
Supplement: Stevens et al. supplementary material 1 — Stevens et al. supplementary material [file S0007125023001526sup001.docx]

*Text message 1: Delivered at 1-month*

Dear [name].

We hope that things have been going well for you since we last had contact.

Just a reminder that the 24-hour contact line (13 11 14) is there if you’d like to connect with someone and Helpline staff (1800 011 511) can put you in touch with your local health service if needed.

Best wishes. [Return SMS messages are unavailable from this service.]

*Text message 2: Delivered at 2, 4, 6, and 10-months*

Hi [name].

We hope that you’ve been ok since our last contact. We’re just checking in with you.

A 24-hour phone line is there for you in case you’d like to connect with someone (13 11 14) or to contact your local health service (1800 011 511).

Best wishes

*Text message 3: Delivered at 3,5,8, and 12-months.*

Dear [name].

Just checking in with you.

A reminder that help is there if you need it. Just call (13 11 14) or (1800 011 511) for support.

Best wishes.
