# Supplementary figures and images for: Efficacy of a short message service brief contact intervention (SMS-SOS) in reducing repetition of hospital-treated self-harm: randomised controlled trial
Source: Br J Psychiatry. 2024 Mar;224(3):106–13. doi: 10.1192/bjp.2023.152 (PMC10884824; doi:10.1192/bjp.2023.152)

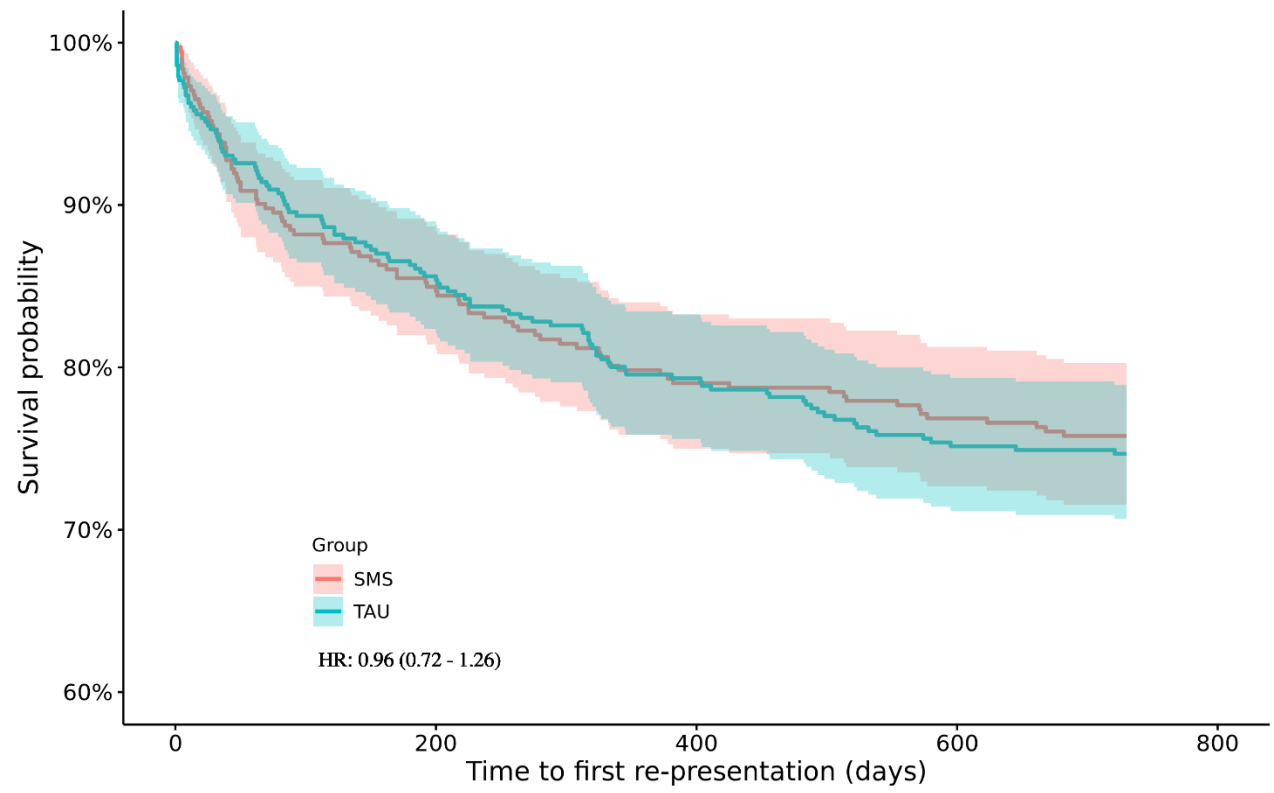

Supplement: Stevens et al. supplementary material 2 — Stevens et al. supplementary material [file S0007125023001526sup002.pdf]
