# Supplementary material for: Efficacy of a short message service brief contact intervention (SMS-SOS) in reducing repetition of hospital-treated self-harm: randomised controlled trial
Source: Br J Psychiatry. 2024 Mar;224(3):106–13. doi: 10.1192/bjp.2023.152 (PMC10884824; doi:10.1192/bjp.2023.152)
Supplement: Stevens et al. supplementary material 3 — Stevens et al. supplementary material [file S0007125023001526sup003.docx]

| Time | Count Model |
| --- | --- |
| 6 months | (RRR = 47%, IRR = 0.53, 95%CI: 0.25 - 1.11) |
| 12 months | (RRR = 37%, IRR = 0.63, 95%CI: 0.34 - 1.17) |
| 24 months | (RRR = 30%, IRR = 0.70, 95%CI: 0.4 - 1.22) |
|  | Zero Model |
| 6 months | (OR = 1.07, 95%CI: 0.72 - 1.59) |
| 12 months | (OR = 0.98, 95%CI: 0.69 - 1.38) |
| 24 months | (OR = 0.94, 95%CI: 0.68 - 1.3) |
