# Supplementary material for: Efficacy of a short message service brief contact intervention (SMS-SOS) in reducing repetition of hospital-treated self-harm: randomised controlled trial
Source: Br J Psychiatry. 2024 Mar;224(3):106–13. doi: 10.1192/bjp.2023.152 (PMC10884824; doi:10.1192/bjp.2023.152)
Supplement: Stevens et al. supplementary material 4 — Stevens et al. supplementary material [file S0007125023001526sup004.docx]

**Supplementary Note 1: Zelen recruitment methodology**

The original Zelen design involves randomization before consent, with consent only required from those allocated to the intervention, whereas the control group receive treatment as usual (TAU) care (1). One of the main features of the original Zelen design is that informed consent is not required from the control group. Baseline characteristics and outcomes are collected from medical records (with ethical approval). However, it is not possible to interact with the control group during follow-up, since they are not informed of their presence in a study. This approach has several benefits. It reduces the burden on patients during clinical presentations, as well as clinicians involved in study enrolments. It also has the benefit of reducing possible bias introduced by the nature of study participation (e.g. additional assessments or monitoring), particularly for those not directly receiving the intervention. A potential drawback of the approach is that only those in the intervention group can withhold consent, potentially leading to unequal sample sizes and possible underestimation or overestimation of effect sizes if these excluded subjects were more or less likely to repeat SH (see Discussion). It is argued that the benefits of the Zelen approach outweigh the risk that it may be considered unethical due to the lack of formal consent. (2,3) Ethics oversight groups must weigh these factors in approving this study design.

1. Zelen M. Randomized consent designs for clinical trials: an update. *Stat Med* 1990; 9(6): 645-56.
2. Carter GL, Clover K, Whyte IM, Dawson AH, D'Este C. Postcards from the EDge: 24-month outcomes of a randomised controlled trial for hospital-treated self-poisoning. *Br J Psychiatry* 2007; 191(6): 548-53.
3. Stevens GJ, Hammond TE, Brownhill S, Anand M, De La Riva A, Hawkins J, et al. SMS SOS: a randomized controlled trial to reduce self-harm and suicide attempts using SMS text messaging. *BMC Psychiatry* 2019; 19: 1-7.
