# Supplementary material for: Efficacy of a short message service brief contact intervention (SMS-SOS) in reducing repetition of hospital-treated self-harm: randomised controlled trial
Source: Br J Psychiatry. 2024 Mar;224(3):106–13. doi: 10.1192/bjp.2023.152 (PMC10884824; doi:10.1192/bjp.2023.152)
Supplement: Stevens et al. supplementary material 5 — Stevens et al. supplementary material [file S0007125023001526sup005.docx]

**Supplementary Note** **2: Systematized Nomenclature of Medicine Clinical Terms (SNOMED CT)**

SNOMED CT is a standardised system of medical terms and diagnoses, developed by the International Health Terminology Standards Organisation ([www.snomed.org](https://protect-au.mimecast.com/s/QY3HCjZ1oBSPEMMyfx1Kjm?domain=snomed.org)) and based in the UK. It allows clinicians to assign diagnoses in the electronic medical record (eMR) of a patient. All Emergency Department (ED) patients in Western Sydney Local Health District hospitals receive a diagnosis with SNOWMED CT, without which they cannot be admitted or discharged from ED. The SNOMED diagnostic system does not always capture SH patients; however, when augmented with the eConsult in the eMR, including a recently introduced reporting field for SH (1), the capture of SH is almost 100% of these case presentations.

1. Bandara P, Page A, Hammond TE, Sperandei S, Stevens GJ, Gunja N, et al. Surveillance of hospital-presenting intentional self-harm in Western Sydney, Australia, during the implementation of a new self-harm reporting field. *Crisis* 2022, Feb 9
